# Polyurethane-Based Composites: Effects of Antibacterial Fillers on the Physical-Mechanical Behavior of Thermoplastic Polyurethanes

**DOI:** 10.3390/polym12020362

**Published:** 2020-02-06

**Authors:** Maurizio Villani, Roberto Consonni, Maurizio Canetti, Federico Bertoglio, Stefano Iervese, Giovanna Bruni, Livia Visai, Salvatore Iannace, Fabio Bertini

**Affiliations:** 1 Istituto di Scienze e Tecnologie Chimiche “Giulio Natta”—CNR, Via A. Corti 12, 20133 Milano, Italy; roberto.consonni@scitec.cnr.it (R.C.); maurizio.canetti@scitec.cnr.it (M.C.); 2 Department of Molecular Medicine (DMM), Center for Health Technologies (CHT), UdR INSTM, University of Pavia, Viale Taramelli 3/B, 27100 Pavia, Italy; federico.bertoglio01@ateneopv.it (F.B.); stefano.iervese01@ateneopv.it (S.I.); livia.visai@unipv.it (L.V.); 3 School for Advanced Studies IUSS, Palazzo del Broletto Piazza della Vittoria, 15, 27100 Pavia, Italy; 4 Department of Occupational Medicine, Toxicology and Environmental Risks, Istituti Clinici Scientifici Maugeri S.p.A Società Benefit, IRCCS, Via S. Boezio 28, 27100 Pavia, Italy; 5 Department of Chemistry, Physical Chemistry Section, University of Pavia, viale Taramelli 16, 27100 Pavia, Italy; giovanna.bruni@unipv.it; 6 Istituto per i Polimeri, Compositi e Biomateriali—CNR, Piazzale Enrico Fermi 1, 80055 Portici (NA), Italy; iannace@unina.it

**Keywords:** polymer matrix composites, thermoplastic polyurethane (TPU), titanium dioxide, silver, chitosan, physical-mechanical properties, antibacterial

## Abstract

The challenge to manufacture medical devices with specific antibacterial functions, and the growing demand for systems able to limit bacterial resistance growth, necessitates the development of new technologies which can be easily produced at an industrial level. The object of this work was the study and the development of silver, titanium dioxide, and chitosan composites for the realization and/or implementation of biomedical devices. Thermoplastic elastomeric polyurethane was selected and used as matrix for the various antibacterial functions introduced during the processing phase (melt compounding). This strategy was employed to directly incorporate antimicrobial agents into the main constituent material of the devices themselves. With the exception of the composite filled with titanium dioxide, all of the other tested composites were shown to possess satisfactory mechanical properties. The best antibacterial effects were obtained with all the composites against *Staphylococcus aureus*: viability was efficiently inhibited by the prepared materials in four different bacterial culture concentrations.

## 1. Introduction

Numerous medical devices inserted into the human body for medical practice can be employed for short periods or remain in place permanently. Once inserted, however, they can create an ideal environment for the development of pathogenic bacteria leading to resilient infections. In that regard, various methods have been proposed to prevent bacterial activity [1]. Among them, the introduction of biocidal fillers directly into the polymeric matrix represents one of the most feasible way to do so [2,3,4]. In this way, it is possible to obtain the desired products without altering the production line and providing a simple way for the realization of the various biomedical probes. Unlike alternative fabrication procedures such as coatings, where it is possible to guarantee a high concentration of antibacterial at the interface, the use of a very effective antibacterial filler does not always guarantee the desired result [1,5,6,7,8,9,10]. In this sense, the choice of a proper antibacterial substance at an adequate concentration becomes decisive, not only for the desired properties, but also to prevent the intrinsic properties of the starting polymeric material from being modified in an inappropriate manner. The excessive increase in the concentration of filler in addition to potentially affecting the thermal and mechanical properties of the bulk material with consequent problems related to their processing, could lead to compatibility problems due to an excessive release of these substances in the body [11,12].

Biomedical devices are usually made of medical grade plastics that are characterized by several requirements, including thermal stability, chemical resistance, long-term durability, aging, and sterilization capability. These qualities are commonly considered to be fundamental for such devices together with a certain resistance to release extractable and leachable materials related to the additives employed in plastics production. Plasticizers, antioxidant, stabilizers, pigments, lubricants, and/or residual monomers lead to compatibility problems and for this reason a series of tests (i.e., cytotoxicity, sensitization, genotoxicity, and hemocompatibility) are carried out on these types of plastics [13]. Moreover, certain types of devices such as catheters, esophageal dynamic stents and ileostomy probes may have complex shapes that cannot be molded directly. For this reason, they are usually made separately and joined together via adhesives or by welding [14,15]. Thermoplastic Polyurethane Elastomers (TPUs) are widely used for catheters and dynamic stents [15,16,17,18,19]. The elastomeric nature derives from its blocky structure whose polymeric chains are composed of alternating sequences of low glass transition (soft) segments and more rigid (hard) segments that soften at higher temperatures. The soft segments are generally polyethers or polyesters and they can influence the thermal properties, weathering resistance, solvent resistance, and mechanical properties. The hard segments are characterized by urethane groups formed by the isocyanate and the chain extender molecules. The rigidity of these segments favors the polymer chains connection, resulting in a three-dimensional crosslinked network responsible for the elastomeric properties. Whether the regularity of these segments could be associated to a partial crystallization or not, the polymer chains connection through secondary interactions—i.e., hydrogen bonding—guarantees a certain degree of physical crosslinking, which prevents the viscous flow of polymeric chains under applied stress. The possibility of varying the hydrogen bonding efficiency by modifying the polymer microstructure or by introducing fillers, which may weaken or strengthen these interactions, can affect the hard/soft domain content. The incompatibility of soft and hard domains forces the phase separation, typical of these materials, and influences some of the TPU properties. However, their elastic properties and their easy processability, typical of thermoplastic materials, make them the perfect candidates for biomedical applications [20,21,22].

Devices which have been designed with antibacterial properties are loaded with fillers, usually belonging to one of the following three families: antibacterial agent release, contact killing, and anti-adhesion/bacteria-repelling [1,3,4,6,7,8,9,10,11,23,24,25,26,27,28,29]. The first two approaches are designed to kill the microbes instead of minimizing their deposition, and usually suffer from a short service lifetime.

Antibiotics are the most effective agents to inhibit bacterial cell wall synthesis by binding to bacterial-enzymes responsible for peptidoglycan crosslinking [19]. Although antibiotics represent a good alternative to potential cytotoxicity problems caused by inorganic fillers, the greatest challenge with antibiotics is bacterial resistance development. The rise of “super-microbes” able to resist many different antimicrobial therapies at the same time prompted new studies and applications related to antimicrobial coatings [1,19].

Similarly, contact-killing agents are able to inhibit bacterial cell walls affecting the cell membrane sterols and/or inhibit some metabolic steps. Silver is one of the few antimicrobial agents capable of killing bacteria even at very low concentrations. Many medical devices can be designed employing silver in the form of ions, alloy, and micro/nanoparticles. Polymer composites containing these fillers are widely used in medical devices approved by international public health organizations. However, each of these silver-containing composites results in more or less efficient antibacterial devices with possible cytotoxicity problems caused by silver release. In this regard, silver nanoparticles represent an interesting way to prevent fast and excessive release of silver [4,8,9,11,12,27].

Antibiotics and contact-killing agents, on the other hand, affect the cell membrane and/or inhibit some metabolic steps, antifouling fillers hamper the adhesion of bacteria on the surfaces preventing the formation of biofilms [1,3,4,6,7,8,9,10,11,23,24,25,26,27,28,29,30,31,32,33]. Generally, they are made of hydrophilic materials or polyzwitterions which are able to minimize the formation of conditioning films via the formation of a hydration layer. The hydration layer, formed through hydrogen bonding and/or ionic solvation, is responsible for steric repulsion, electrostatic repulsion, and low surface energy [23,24,25,26,30,31,32].

The object of this work is the study and development of innovative materials for the realization and/or implementation of biomedical probes with specific antibacterial functions for specific insertion durations. We used a medical grade TPU as a polymeric matrix filled with different additives chosen for their known antibacterial characteristics. We selected silver and titanium dioxide fillers for their contact killing ability and chitosan for its recognized antifouling effect, especially in combination with other polymers such as polyvinylalcohol [30]. These antibacterial agents were introduced through simplified processing technique easily scalable to industrial level, for the realization of biomedical probes. Although these fillers have already been used as antimicrobial agents in consumer products and reported in the literature, the preparation and characterization of the composites provides us with interesting information on the fillers effect on TPU physical-mechanical properties in view of the simplified processing technique used. A further study of the use of these fillers in the form of coatings will be presented in a subsequent paper, allowing for a direct comparison between the alternative preparation techniques (i.e., melt processing and post-processing).

## 2. Materials and Methods

### 2.1. Materials

The Estane^®^ 58887 NAT 036, a thermoplastic polyurethane (TPU) of biomedical grade characterized by an excellent resistance to hydrolysis with excellent performance at low temperature and clarity, was supplied by Lubrizol (Wickliffe, OH, USA). Micrometric silver powder (Ag) with a size distribution ranged from 2 to 3.5 µm, titanium dioxide anatase nanopowder (TiO_2_) with a size distribution ≤25 nm and low molecular weight chitosan (Chit) with an average molecular weight of 50–190 kg/mol (based on viscosity) and a degree of deacetylation of 75–85%, were purchased by Sigma-Aldrich, Milano, Italy.

### 2.2. Polyurethane-Based Composite Preparation

TPU-based composites were prepared by melt compounding, using a Brabender electronic plasticorder AEV 153 mixer at 190 °C by applying a rotor speed of 60 rpm for 5 min. Before compounding, the polymer matrix was dried at 105 °C for 5 h. During processing, dry nitrogen was continuously purged into the mixing chamber. Neat TPU was treated under identical conditions for reference purposes. We prepared composites with 5 wt % of filler concentration, since well dispersed materials with reasonable antibacterial properties were obtained at this concentration for all the fillers. The materials thus obtained were die-cast with a heated plate press, to obtain 250 µm-thick films with a controlled geometry using the following procedure: (i) heating at 190 °C for 5 min at 5 bar; (ii) heating at 190 °C for 5 min at 20 bar; and (iii) cooling to room temperature by cold water.

### 2.3. Characterization

^13^C and ^1^H NMR spectroscopy was applied to investigate the microstructural characteristics of the TPU sample. Spectra have been recorded at 600.13 and 150.90 MHz for ^13^C and ^1^H nuclei respectively, at 70 °C in tetrachloroethane solvent.

Size exclusion chromatography (SEC) was performed using a Waters (Milford, MA, USA) GPC600 chromatographic system with refractometer detector, in tetrahydrofuran (THF) at 35 °C. The column set was composed of three columns (Polypore, Oligopore and 50 Å) from Polymer Laboratories. The SEC system was calibrated using polystyrene standards.

Wide angle X-ray diffraction (WAXD) data were obtained using a Siemens D-500 diffractometer equipped with a Siemens FK 60-10 2000W tube (Cu Kα radiation, *λ* = 0.154 nm). The operating voltage and current were 40 kV and 40 mA, respectively. The data were collected from 5 to 50 2θ° at 0.02 2θ° intervals.

Small angle X-ray scattering (SAXS) measurements were conducted with a Kratky Compact Camera. Monochromatized Cu Kα radiation (*λ* = 0.154 nm) was supplied by a stabilized Siemens Krystalloflex 710 generator and a Siemens FK 60-10 2200 W Cu target tube operated at 40 kV and 45 mA. The scattered intensity was counted in different ranges of 2θ°, by using a step scanning proportional counter with pulse height discrimination, the abscissa variable was h = sin(θ) 4π/*λ* the data were successively corrected for blank scattering and desmeared.

Thermogravimetric analysis (TGA) were performed on a PerkinElmer (Waltham, MA, USA) TGA 7 instrument at a scan rate of 20 °C/min under air. TGA and derivate thermogravimetry (DTG) curves were recorded from 100 up to 750 °C.

Differential scanning calorimetry (DSC) analysis was carried out under nitrogen flow using a PerkinElmer (Waltham, MA, USA) DSC 8000 calorimeter. Before DSC scans, the materials were annealed at 105 °C for 5 h. The samples were heated from −70 to 240 °C at a rate of 20 °C/min and kept at 240 °C for 3 min to erase previous thermal history. Then they were cooled to −70 °C at 20 °C/min and subsequently heated with the same rate up to 240 °C.

Contact angle measurements were performed using a CAM 200 from KSV Instruments Ltd. (Helsinki, Finland). Typically, a 10 μL drop of water was deposited on the surface of each prepared film to determine the static contact angle. Drops of brain heart infusion (BHI, Formedium, Norfolk, UK) and Luria Bertani broth (LB, Scharlau, Barcelona, Spain) were equally deposited to compare the surface wettability of the prepared films by using the bacterial mediums employed for antibacterial tests. The data were averaged by a series of five measurements per sample.

To capture high-resolution images and obtain elemental maps of the atomic elements of the composites, a Zeiss EVO-MA10 scanning electron microscope (Carl Zeiss, Oberkochen, Germany) coupled to an energy dispersive X-ray spectroscopy (EDS) detector (X-max 50 mm^2^, Oxford Instruments, Oxford, UK) was utilized. Acceleration voltage used was 20 kV. Samples were not gold sputtered prior to EDS analysis.

To assess the mechanical properties of the materials, static tensile tests were performed at room temperature using a Zwick-Roell (Ulm, Germany) Z010 dynamometer with a 50 N load cell at a speed of 5 mm/min until the specimen broke. Dumbbell shape specimens with an overall length of 75 mm, a gauge length of 25 mm and a width of narrow section of 4 mm were cut from compression molded sheets (thickness about 0.25 mm). At least five specimens were tested for each individual sample.

The rheological behavior of the samples was analyzed in a dynamic regime using a rotational rheometer AR 2000 from TA Instruments (New Castle, DE, USA). Frequency sweep tests were carried out in the range between 0.04 and 628.3 rad/s at a temperature of 190 °C and 1% strain amplitude using parallel plates with a diameter of 25 mm and with a gap of around 0.5 mm. Temperature sweep experiments were conducted at 10 rad/s in the temperature range 160-220 °C at a rate of about 0.7 °C/min.

### 2.4. Antibacterial Tests

*Escherichia coli* ATCC 25922 (*E. coli*) and *Staphylococcus aureus* ATCC 25923 (*S. aureus*) were used in this study as main representatives of Gram-negative and Gram-positive bacteria. *E. coli* was routinely grown in Luria Bertani Broth (LB) and *S. aureus* in Brian Heart Infusion (BHI) overnight under aerobic conditions at 37 °C, 200 rpm (Certomat^®^ BS-T, B.Braun Biotech International, Melsungen, Germany). To evaluate the antimicrobial activity of TPU-based composite films, the overnight cultures were diluted in a fresh appropriate medium and 200 µL of diluted bacterial suspension were deposited on TPU composites film discs placed at the bottom of a 96-well flat-bottom polystyrene tissue culture plates (TCP) well. Prior to any use, the materials were sterilized with 70% ethanol and well rinsed in a phosphate buffered saline (PBS). Different bacterial concentrations were tested to evaluate the effect of filler towards increasing inocula, ranging from 1 × 10^2^ up to 1 × 10^5^ bacterial cells/mL. These diluted suspensions were obtained by comparing the optical density at 600 nm (OD_600_) of the overnight culture with a standard curve correlating OD_600_ to cell number. The TCP was then incubated at 37 °C in static conditions. Furthermore, control wells of TCP were used as positive and sterility control. At the end of the culturing period, the bacterial viability was assayed through the quantitative 3-[4,5-dimethylthiazol-2-yl]-2,5diphenyltetrazoliumbromide (MTT) (Sigma Aldrich, St. Louis, MO, USA) test [32,33]. 5 mg/mL of MTT solution, dissolved in PBS, was used as stock solution and the working concentration was 0.5 mg/mL. The test was performed at 37 °C for 3 h. Upon presence of viable cells, reduction of the MTT salt results in purple insoluble formazan granules. These precipitates are dissolved through acidified 2-propanol (0.04 N HCl). The result was recorded through a Clariostar^®^ microplate reader (BMG-Labtech, Ortenberg, Germany) at 570 nm with the reference wavelength set at 620 nm. Cell survival was expressed as percentage of the number of bacteria survived on TPU composites to number of bacteria grown on neat TPU, the latter set at 100%. Experiments were conducted in triplicate and repeated twice.

SEM images of *S. aureus* grown on TPU films were prepared essentially as already reported [33]. Briefly, bacteria were incubated on sterile neat and composite TPU film for 24 h at 37 °C. Following incubation, samples were washed carefully with PBS and fixed with 2.5% (v/v) glutaraldehyde in 0.1 M Na-cacodylate buffer (pH 7.2), for 1 h at 4 °C. After additional washing with cacodylate buffer to remove the excess of glutaraldehyde, the samples were dehydrated using increasing concentrations of ethanol (25, 50, 75%) for 5 min and final two washes of 10 min in 96% ethanol. The samples were then lyophilized for 3 h using an Emitech (Ashford, UK) K-850 apparatus and placed on a mounting base. Finally, TPU discs were sputter coated with gold (300 nm) and investigated using a Zeiss EVO-MA10 scanning electron microscope (Carl Zeiss, Oberkochen, Germany), acceleration voltage used was 20 kV.

## 3. Results and Discussion

### 3.1. Preparation, Molecular, and Structural Characteristics

Polyurethane-based composites, containing a fixed concentration (5 wt %) of Ag, TiO_2_ and chitosan, were prepared through polyurethane melt processing. In this way, it was possible to introduce antibacterial charges at the compounding phase, providing an interesting alternative, easily scalable at an industrial level, for the manufacture of biomedical probes.

**Figure 1:**
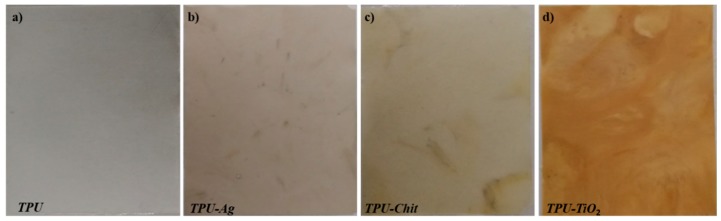
Compression molded films (dimensions 80 × 115 mm, thickness 0.25 mm) of TPU (**a**), TPU-Ag (**b**), TPU-Chit (**c**) and TPU-TiO_2_ (**d**).

The obtained composites were compression molded and homogenous films were obtained (Figure 1). Small color variations associated with the filler loading were observed; only the sample filled with TiO_2_ shows a more pronounced yellowing probably due to some polymer degradation phenomena [6].

**Figure 2:**
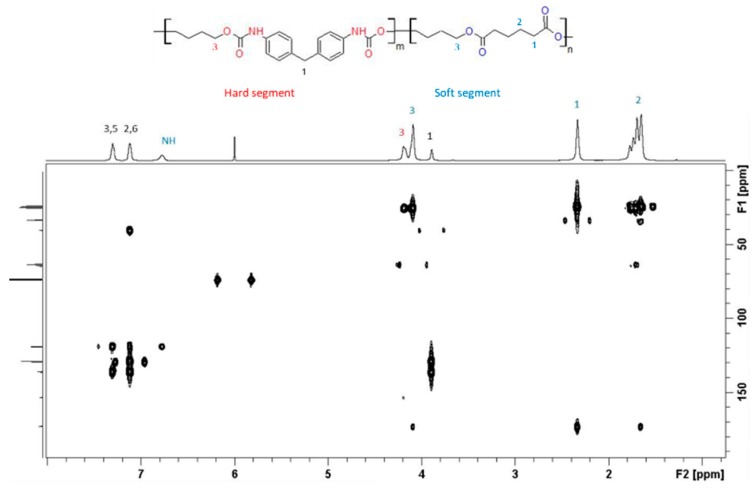
Bidimensional heteronuclear ^1^H-^13^C spectra of TPU.

NMR spectra recorded on neat TPU are reported in Figure 2. NMR signal assignment and structure correlation is reported in Part S1 of Supplementary Materials.

To better understand the influence of the thermo-mechanical treatment we measured, the molecular weight of two thermoplastic polyurethanes homopolymers with different thermal history, i.e., before and after the processing. From the values reported in the Table 1, a molecular weight reduction is observed following the mixing treatment, which can be associated to the hydrolysis of the polyurethane chains due to some water absorption during the compounding [34]. Although these materials were previously conditioned according to the procedure described in Section 2.2 and a nitrogen flow was directed towards the mixing chamber, this did not completely guarantee the absence of water and excluded a possible water uptake during the processing itself.

In addition, the presence of the fillers affected the molecular weights. The samples loaded with chitosan and Ag present a very slight reduction of the average molecular weights (lower than 5%) compared to processed TPU. This reduction becomes noticeable (about 30%) for TPU-TiO_2_ sample. Titanium dioxide, particularly in the form of anatase, exhibits photocatalytic activity. This affects the polymer degradation due to the formation of electron–hole pairs that may generate free radicals (e.g., hydroxyl radicals) which are capable of degrading the polymeric matrix [6]. All samples show a similar narrow polydispersity (*M*_w_*/M*_n_).

**Figure 3:**
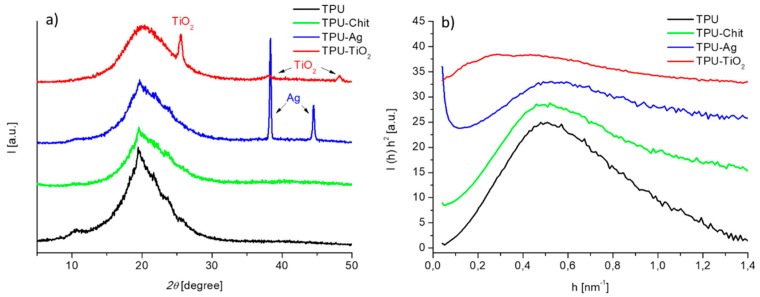
WAXD (**a**) and SAXS (**b**) profiles of TPU and TPU-based composites.

Figure 3a shows the WAXD profiles of TPU and TPU composites. By comparing the reported profiles, evident analogies among the samples could be observed. The data confirm that as widely reported in literature for TPU, these samples are characterized by the presence of ordered structures in an amorphous matrix [35]. However, TPU presents stronger small sharp peaks at the top of a broad amorphous halo compared to the composites and these signals are displayed in the 2Ɵ ranges 9–11° and 20–30°. The diffraction peak at 2Ɵ = 10° gives information about the unit cell projection along the *c* axis while the peak at 20° represents the interchain distance between two chains lying in the *ac*-plane and spaced by hydrogen bonds [36]. Finally, the peaks in the range 21–25° give information about the distance among adjacent hydrogen bonded sheets (intersheet distance) [36,37]. The intensities of these peaks reduce for TPU-Chit and TPU-Ag until disappearing for TPU-TiO_2_. These results suggest that a less ordered crystalline structure attributed to hard domains presence is promoted by introducing the fillers employed. The effect of TiO_2_ is the most pronounced in that sense and this could also be related to the complementary degradative effect on TPU matrix observed for such a composite. The TPU-Ag and TPU-TiO_2_ WAXD profiles also present narrow diffraction peaks of the inorganic fillers.

The SAXS Lorentz-corrected desmeared curves of TPU and TPU-based composites are reported in Figure 3b. The models that are normally applied to two-phase segmented polyurethanes consider that the scattering results from the electron contrast between hard and soft phases [38,39,40]. The profile of the TPU sample exhibits a peak corresponding to a mean interdomain distance (or long period, L) of 12.6 nm; similar profile was registered for the TPU-Chit composite where a L value of 12.8 nm was calculated. The profile of the TPU-Ag composite exhibits an intense scattering at low angles due to the presence of Ag particles and a reduced intensity scattering of the TPU matrix suggesting a less ordered structure. The very broad profile registered for the TPU-TiO_2_ composite suggests a marked destruction of the ordered structures.

### 3.2. Thermal Analysis

**Figure 4:**
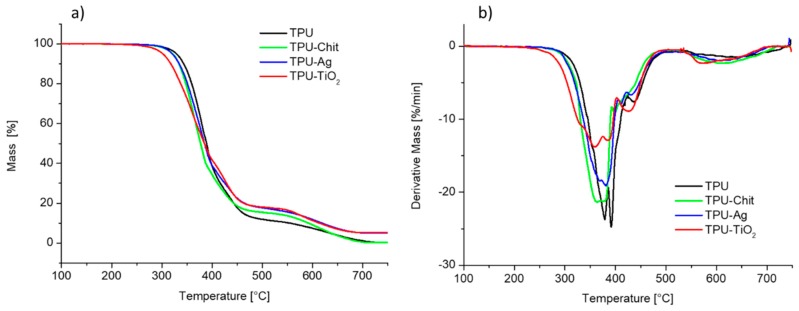
TGA (**a**) and DTG (**b**) curves for TPU and TPU-based composites.

TGA experiments were performed to determine the thermal stability of TPU and TPU-based composites (Figure 4). All the experiments were performed under oxidative atmosphere, to check whether the residual amount of the inorganic fillers could confirm the nominal amount employed during the compounding. A mass residual of about 5% was observed for the TPU composites with Ag and TiO_2_ at the end of the thermal cycle (750 °C), whereas the homopolymer and the sample filled with chitosan did not present any residue at this temperature as expected. Table 1 summarizes the TGA experimental data including the onset of degradation, taken as the temperature at which 2% mass degradation occurs (*T*_2%_) and the temperature corresponding to 50% mass loss (*T*_50%_).

The thermo-oxidative degradation of neat TPU takes places in successive decomposition steps occurring between 280 and 700 °C. According to the literature, the first degradation stage is ascribed to the decomposition of the hard segment with the formation of di-isocyanates and diols, whereas the second step is due to the decomposition of the soft segments [41,42]. The last step from 520 to 700 °C is due to the oxidation process of the carbonaceous residue formed in the previous steps. The shapes of the TGA curves for the TPU-based composites are similar to that observed for the neat TPU. We note, however, that the composites showed differences in the values of onset and 50% mass loss degradation. In particular, the presence of TiO_2_ gives rise to a *T*_2%_ decrease of about 30 °C mainly due to the photocatalytic activity of this filler and its effect on the polymer molecular weight (see Section 3.1). On the contrary, TPU-Chitosan and TPU-Ag present *T*_2%_ similar to the neat TPU. The *T*_50%_ values range from 379 to 386 °C, nevertheless a close inspection of the DTG curves highlighted some differences in the multi-steps thermo-oxidative degradation. For example, TPU-TiO_2_ composite shows a lower mass loss rate in the degradation range between 330–400 °C, followed by a faster degradation step at higher temperatures (400–480 °C), compared to TPU and the other TPU-based composites.

TPUs are multi-block copolymers consisting of polar hard and less polar soft segments, and their thermodynamic incompatibility promotes the phase separation [43]. Therefore, TPUs final properties can be related to the amount of the hard and soft segments and controlling the morphology is essential to obtain the desired product properties [44]. In this scenario, thermal history is determinant for the micro-aggregation, structure, and properties of TPUs, especially in the presence of well-organized hard segment domains capable of crystallizing under opportune conditions. Many works have shown multiple thermal transitions of TPUs [40,45,46,47,48]. These transitions are generally the following: (i) glass transition (*T*_g_) of either the hard or soft domains; (ii) endotherms associated with the melting of crystalline hard and/or soft domains; and (iii) endotherms associated with the melting of annealed crystalline hard domains. These endotherms are related to possible different morphologies of the crystallizable segments very sensitive to thermal/processing history [21,48].

**Figure 5:**
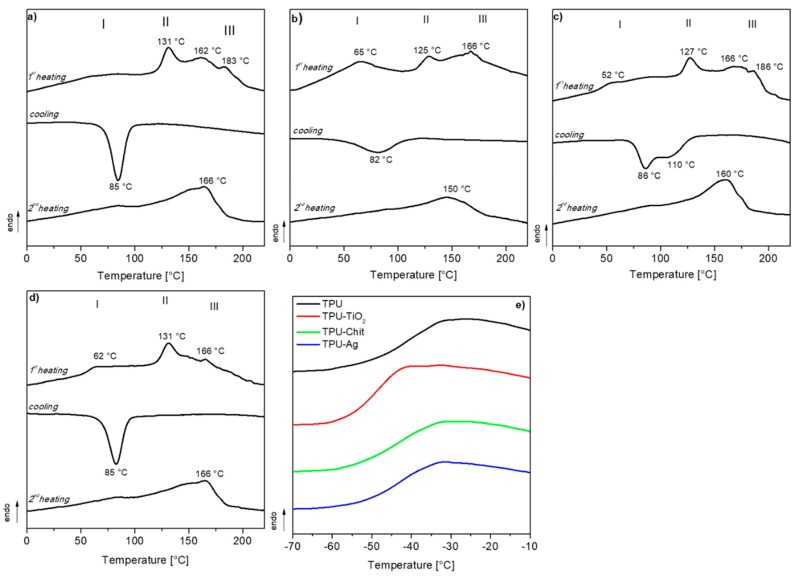
DSC traces (first heating, cooling and second heating) of TPU (**a**), TPU-TiO_2_ (**b**), TPU-Chit (**c**), and TPU-Ag (**d**); *T*_g_s of TPU and TPU-based composites from first heating run (**e**). Three endotherm regions (I, II, III) are visible and related to different morphologies of the crystallizable segments.

Figure 5a–d reports the DSC traces, i.e., the first heating, cooling scan and the second heating, for the samples previously subjected to a thermal annealing at 105 °C for 5 h. Similarly to what was reported in [21,47], multiple endotherms are visible for all the samples. The I and II endotherms represent the melting of hard segments with a relatively short-range order. In contrast, the III endotherms observed at a higher temperature range (around ca. 180 °C) are attributed to the melting of well-organized hard domains due to aromatic polyurethane segments. In general, the position and the shape of the endotherms depends on the length and distribution of the crystalline segments along the polymer chain. We were, however, able to observe differences related to the shape and position of the endotherms, due to the filler’s presence, when comparing the first heating run of TPU and TPU-based composites (Figure 5a–d). Neat TPU exhibits three endotherms with peaks at 131, 162, and 183 °C in the regions II and III of the DSC curve, while no significant endotherm due to the short-range order domains are visible in region I (see also Figure S2, Supplementary Materials). The presence of TiO_2_, chitosan and Ag promotes the crystallization of such disordered structures, which exhibit melting peak (*T*_m_) at 65, 52 and 62 °C, respectively. The samples were cooled at scanning rate of 20 °C/min from homogeneous melt conditions (240 °C for 3 min) and the crystallization events were registered. Narrow crystallization peak centered at 85 °C is observed for TPU and TPU-Ag, while TPU-TiO_2_ presents a broader, less intense exothermic peak at about 82 °C. A broad crystallization event characterized by a peak centered at 86 °C and a shoulder at high temperature (about 110 °C) is observed for TPU-Chit. The second heating runs show similar trends for all the samples, as an effect of the new thermal history imparted on the materials during the non-isothermal crystallization step. A broad and poorly defined endotherm with a melting peak at about 165 °C was registered for all the samples, with the exception of TPU-TiO_2_ for which this event lowers to 150 °C. The glass transition events of the soft segments for TPU and TPU-based composites are shown in Figure 5e. Their respective *T*_g_ values range between −45 and −40 °C, with the exception of TPU-TiO_2_, which presents a *T*_g_ of about −50 °C as expected for a material with reduced molecular weight.

### 3.3. Surface Morphology

**Figure 6:**
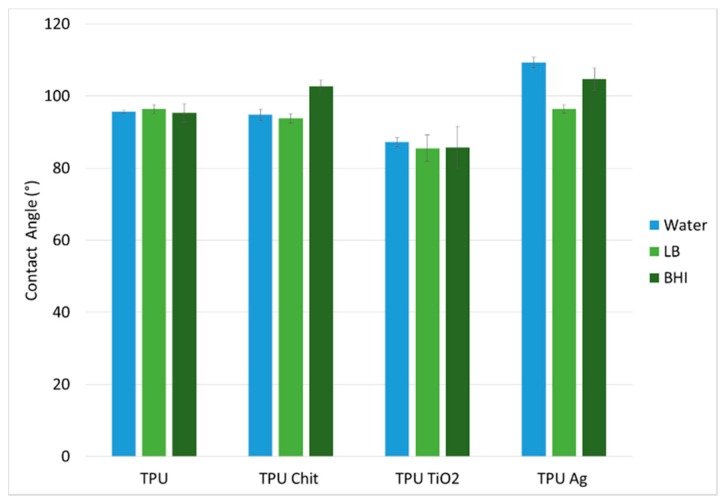
Average contact angle values in water, Luria-Bertani broth (LB) and brain heart infusion (BHI) for TPU and TPU composites.

To study the surface morphology of the prepared samples and determine the presence of each filler employed, different characterization techniques were used. Firstly, the wettability of the composites was investigated and compared to neat TPU. In particular, static contact angle measurements were performed using water, Luria-Bertani broth (LB) and brain heart infusion (BHI), the two bacterial culture mediums employed for bacterial growth. The equilibrium contact angle of a drop of water on an ideal surface conventionally quantifies the wettability of a solid by a liquid. By convention, surfaces having an angle of contact (θ) with water greater than 90 degrees are defined as hydrophobic and surfaces with θ less than 90 degrees are hydrophilic. From the contact angle data shown in Figure 6, it is possible to observe the predominantly neutral or slightly hydrophobic nature of the TPU, TPU-Chit, and TPU-Ag composites surfaces for all the three liquids. On the contrary, the presence of TiO_2_ imparts a slightly hydrophilic nature (θ ≈ 85°), observed for all the liquids used. The partial degradation of the polymer matrix caused by this filler is associated with an increase in the oxidized functional groups responsible for the hydrophilic nature observed.

SEM measurements were carried out to further investigate the presence of fillers on the surfaces of the melt-compounded film. Morphologically, the films appear quite homogenous, with some surface irregularities, most probably related to the compression molding procedure. As shown in Figure S3 (Supplementary Materials), no apparent differences can be distinguished nor is the presence of fillers observed, since these surfaces were not previously coated with any metal.

**Figure 7:**
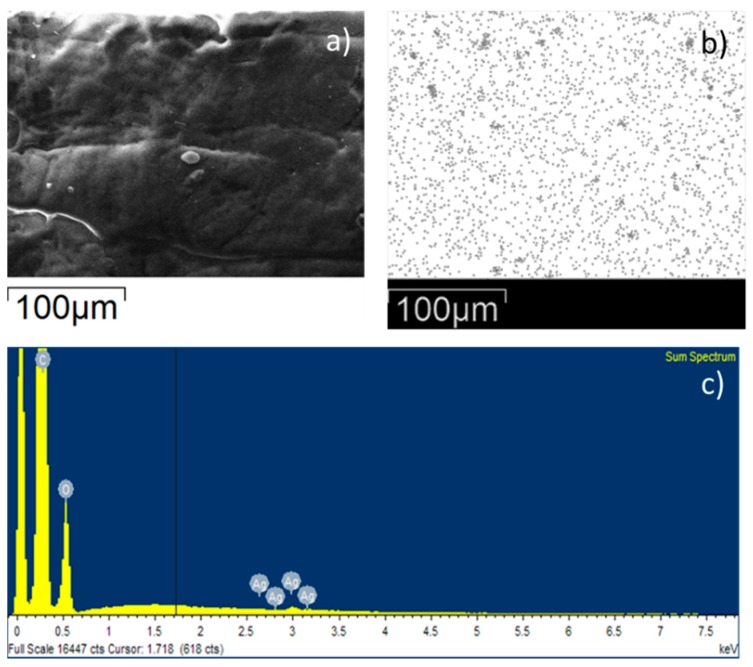
SEM image of TPU-Ag (**a**), map showing the relative position of Ag on the surface (**b**), and EDS spectrum for TPU-Ag film (**c**).

**Figure 8:**
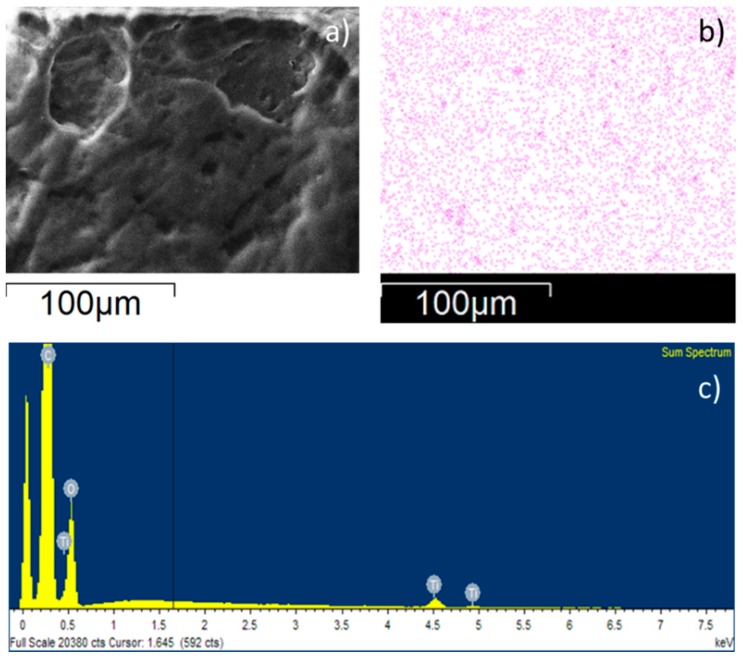
SEM image of TPU-TiO_2_ (**a**), map showing the relative position of TiO_2_ on the surface (**b**), and EDS spectrum for TPU-TiO_2_ (**c**).

In addition, the elemental analysis of Ag and TiO_2_ atoms by SEM-EDS highlighted a homogeneous distribution of the fillers on the polymeric surfaces (Figure 7 and Figure 8). SEM images, the corresponding map showing the relative position of Ag and TiO_2_ and the EDS spectra are presented in Figure 7a–c; Figure 8a–c, respectively. SEM-EDS inspections on TPU materials containing Ag and TiO_2_ reveal that 0.25% and 0.63% of the fillers are respectively present. The fractions of Ag and TiO_2_ were calculated considering the EDS sum spectrum measured on sample film of 300 × 250 µm.

Relative weight % of the elements detected in both materials are summarized in Table S1 (Supplementary Materials).

### 3.4. Mechanical and Rheological Properties

**Figure 9:**
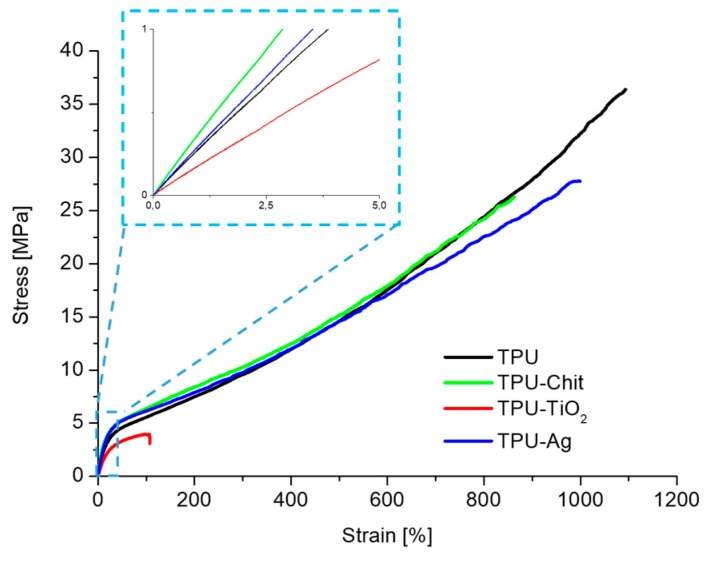
Stress–strain curves for TPU and TPU-based composites. The inset picture highlights the initial part of the stress-strain curves.

The mechanical tensile behavior of the studied materials was evaluated at 20 °C by uniaxial stretching until failure. The stress–strain curves are reported in Figure 9 and the main tensile properties, namely elastic modulus (*E*), maximum stress (*σ*_max_), and elongation at break (*ε*_break_) are summarized in Table 2.

Neat TPU shows the typical mechanical behavior of the thermoplastic elastomeric polyurethanes for which the thermodynamic incompatibility between the hard and the soft segments and the resulting phase separation is responsible for the well-known flexibility and toughness [48]. The addition of 5 wt % TiO_2_ to the polymer matrix results in an evident decrement of the tensile modulus, accompanied by a considerable reduction in tensile strength and elongation at break. These drastic reductions are related to the loss of crystalline ordered structures (evidenced by X-ray techniques) responsible for the elastomeric properties of TPUs (see the rheological part) and to the partial degradation of the polymeric matrix following processing, as evidenced by the molecular weight data reported in Section 3.1. The addition of chitosan and Ag generates a slight increase in the rigidity of the material, as confirmed by the higher elastic modulus. A reduction of the maximum tensile strength and elongation at break is also observed, highlighting a general decrease in the ductility of these materials.

Rheology is widely used to study polymer composites flow-properties, since their behavior is in between those of pure polymeric melt and colloidal suspensions. Consequently, knowledge of the viscoelastic properties of filler/polymer composites and their comparison with the homopolymer, is of basic importance for their processing.

**Figure 10:**
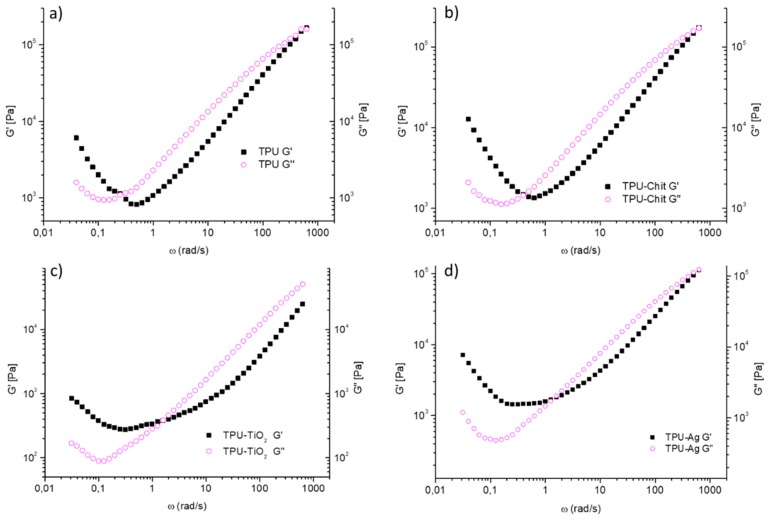
Storage modulus (G’) and loss modulus (G’’) as function of the frequency range ω = 628.3–0.04 rad/s at 190 °C for TPU (**a**), TPU-Chit (**b**), TPU-TiO_2_ (**c**), and TPU-Ag (**d**).

The rheological properties of TPU and TPU composites were investigated using a stress-controlled rheometer at 190 °C and Figure 10 shows the frequency dependence of the storage modulus (G’) and the loss modulus (G”) at constant temperature. The experiments were performed starting from high angular frequencies (ω = 628.3 rad/s) and going to low frequencies (ω = 0.04 rad/s). In all the curves reported here, the viscous behavior prevails in the initial part of the experiment at high angular frequencies (G”> G’) while the elastic behavior prevails at the end of the experiment at low angular frequencies (G’> G’’). However, we did not register the typical 2–3 orders of magnitude difference between G” and G’ values in their melting state, where the viscoelastic behavior dominates. Both moduli decrease as a function of the reduced angular frequency, starting from a first crossover at high angular frequencies to a second crossover, which occurs in the frequency range of 0.1–1 rad/s. Consistently with the measured molecular weight of TPU and TPU composites, the first crossover of TPU, TPU-Chit, and TPU-Ag is in the same ω range. The low molecular weight of TPU-TiO_2_ makes this point to fall out of the experimental limits of the instrumentation. The second crossover point at low frequencies is registered for all the samples and represents the instant in which the material goes from a gel/liquid like behavior to a pseudo-solid like behavior where both moduli increase monotonically, but G’ rises more markedly than G” [49,50,51,52,53,54]. As well-known from the literature a sol/gel transition is designated by the storage modulus and the loss modulus running in parallel to each other in a double logarithmic plot as a function of angular frequency [51,53,54]. By recording G’ and G’’ vs. frequency, we observed that the two moduli decrease (with an analogous trend), approach and pass each other as reported elsewhere for TPUs and confirming a gel like behavior [51]. Comparable trends were observed for all the samples investigated, suggesting the coexistence of well-organized hard crystalline domains embedded in the molten polymer matrix (see Region III, DSC data). This phase separation has a strong influence on the rheology of the system and the interactions among solid domains are responsible for the pseudo-solid like behavior at low ω (from 0.04 to 1 rad/s), attributed to the formation of three dimensional network structures and usually observed when fillers are dispersed in a polymer matrix [55]. In particular, remarkable shear thinning behaviors have been reported for composites where nanofillers were embedded in a polymer matrix, especially by increasing the filler loadings [56]. In our case, this behavior is observed for the homopolymer itself and therefore, attributed to the formation of hydrogen bonded network-like structures that reduced the mobility of polymer chains. For this reason, a similar G’ trend at low frequencies, associated to hydrogen bonding efficiency, is observed for TPU-Chit and TPU-Ag as well. On the contrary, a significant deviation of G’ is registered for TPU-TiO_2_ probably due to a perturbation of hydrogen bonding caused by this filler. However, the dimensions and shape of these polymer–polymer hard domain networks are affected by the fillers, which in some way influence the efficiency of the secondary bonding responsible for such a network formation. For this reason, a crossover shift from values of ~0.2 rad/s observed for TPU to higher angular frequencies of TPU-Chit (~0.3 rad/s), TPU-Ag (~1 rad/s), and TPU-TiO_2_ (~1 rad/s) was observed. This confirms that shorter relaxation times are required to respond to the applied solicitation, typical of smaller domains.

The observed increment of moduli, registered for all the samples at low ω (from 0.04 to 1 rad/s), differs from what is reported in literature, where a decrease or plateau is usually reported [49,55]. Our hypothesis is that the observed phenomena are related to crystallization of hard domains of the TPU, under the employed conditions and the different filler used. Frequency sweep experiments were performed at 190 °C, which is not far from the DSC region III (around 180 °C) where endotherms attributed to the melting of well-organized hard domains were observed. To confirm our hypothesis, time sweep experiments at 190 °C at three different angular frequencies (0.1, 1, and 10 rad/s) were performed on TPU (see Figure S4, Supplementary Materials). An increment of moduli values during time evolution is registered with all the three angular frequencies used. However, elastic behavior prevalence is observed along the whole experiment, only for TPU measured at ω = 0.1 rad/s, or rather, when longer relaxation time allows polymer chains to crystallize easier. Moreover, the same experiment at ω = 0.1 rad/s was performed on TPU-TiO_2_ as well. A similar trend, characterized by lower values of moduli along the whole range of time, was observed. The data strengthen our hypothesis on the coexistence of well-organized hard crystalline domains embedded in the molten polymer matrix and confirm that TiO_2_ affects the most the TPU crystallization, justifying the less pronounced increase at low frequencies shown in Figure 10c.

Differently from the typical behavior ascribed to the filler-filler interactions, which are usually responsible for pseudo-solid like behavior as function of filler concentration, their use here seems to negatively affect the well-known microphase separation of TPU. Probably a further filler increase could promote an even higher increase of G’ and G” at low angular frequencies, once the interactions among fillers become dominant over TPU hydrogen bonding [57]. However, the data reported here allow us to explain the 3D network formation of hard domains and microphase separation occurring at these temperatures.

**Figure 11:**
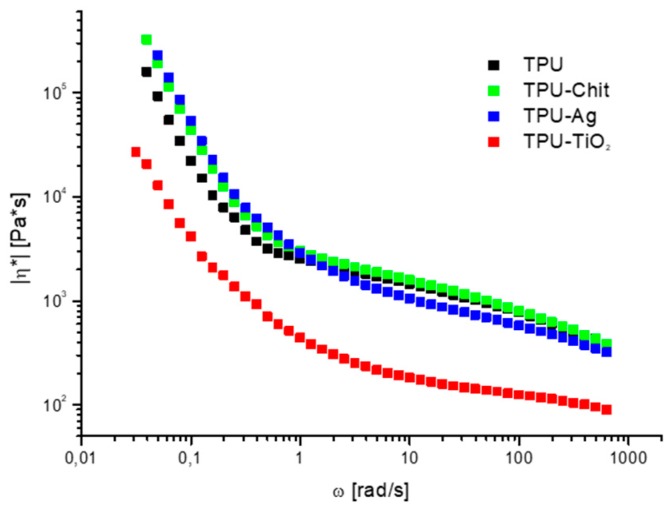
Complex viscosity as function of the frequency range ω = 628.3–0.04 rad/s at 190 °C for TPU and TPU-based composites.

Figure 11 shows the dependence of complex viscosity on ω for the sample series prepared. A similar trend of complex viscosity is registered for TPU, TPU-Chit and TPU-Ag. Their values are higher than those of TPU-TiO_2_ in the whole range of frequencies. In particular TPU-Ag and TPU-Chit at low frequencies, show an increment η*, while the same behavior of the TPU is observed at high frequencies (1.5–628 rad/s). It seems probable that Ag helps polymer chains to slip at high frequencies, promoting disaggregation of the hard domains associated with the observed decrease in viscosity. The addition of chitosan, characterized by many functional groups involved in the hydrogen bonding, does not alter this trend and a similar viscosity behavior to TPU is observed. Similarly to what mentioned above for G’ and G” trends, the limits of the two different viscosity regimes observed for each sample change as function of the hard domains shape and dimensions. The addition of TiO_2_ alters the pseudo-solid like behavior reducing the dimensions of the hard domains reflected in a smoothing of the curve reported in Figure 11. Again, as was observed for TPU-Ag, TiO_2_ promotes polymer chains slipping at high frequencies. Moreover, the degradation of the polymeric matrix associated with the presence of TiO_2_ (see Section 3.2), causes a reduction of the viscosity along the whole range of frequencies.

**Figure 12:**
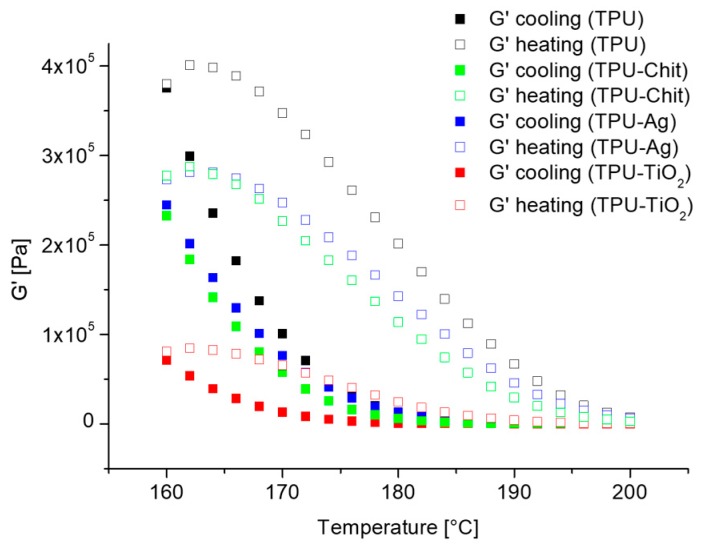
G’ at 10 rad/s as a function of the temperature range *T* = 160–200 °C, on cooling (filled symbols) and on heating (open symbols) for TPU and TPU composites.

To determine whether or not the hydrogen bonding presence serves to increase the overall cohesion of these hard domains and more in general the properties of these materials, G’ and G” were measured at a constant frequency during a cooling/heating cycle. It is important to note, as reported by Seymour and Cooper, that there is still significant hydrogen bonding at 200 °C [58], which favors the formation of the network structure responsible of the registered phase separation and pseudo-solid like behavior. The 3D network of hard domains slows down the relaxation and this is shown in Figure 12 where the temperature dependence of the storage modulus G’ at 10 rad/s for TPU and TPU composites during cooling from 200 to 160 °C (filled symbols) and heating vice versa (open symbols) is shown. In thermoplastic elastomers, the amplitude of the oscillations of the lattice elements of the crystalline domains increases by temperature increasing. As a result, G’ decreases with temperature [59]. The storage modulus deviation observed, can be directly related to the degree of crystallization when measuring during crystallization and especially to analyze physical gels behavior [60,61]. Although many authors have suggested using mild conditions (i.e., at 1 rad/s and a strain amplitude of 0.5%) to prevent flow induced crystallization phenomena, we selected an angular frequency of 10 rad/s, to avoid the second crossover of many tested materials [60,62]. We believe that this choice did not influence the observed trend, at most it influenced the value of the deviation measured for each sample.

In the case of TPU, during cooling, the storage modulus increased rapidly when micro-phase separated, deviating from the linear trend expected in a log scale for a change in temperature [63] (Figure S5, Supplementary Materials). This larger increase in G’ is found for the neat polymer where the hard domains network are bigger in dimensions and characterized by an unperturbed hydrogen bonding efficiency.

During heating, a different G’ trend is registered due to the melting of these domains that occurs at a temperature higher than they require to form on cooling. The linear dependence of G’ on temperature is only restored above 195 °C, when a homogeneous melt is obtained. A similar trend is also observed for the TPU composites. However, since the hydrogen bonding efficiency is perturbed by the filler presence and the hard domains size reduce, G’ deviation in these cases is smaller. In particular, both crystallization and melting shift to lower temperature compared to neat TPU as effect of the reduced and less defined crystals obtained. TPU-TiO_2_ consistently with the previous data, affects the most this behavior. Supplemental information regarding the temperature sweep experiments are reported below (Appendix A).

### 3.5. Antibacterial Properties

**Figure 13:**
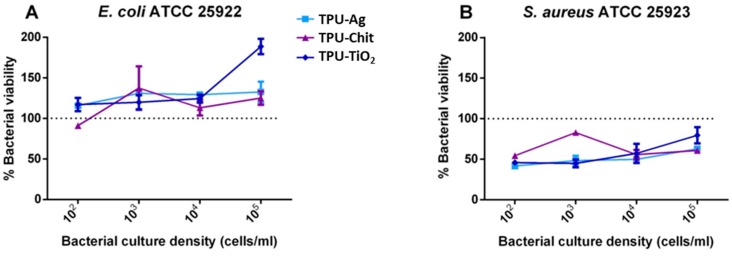
Antibacterial activity of TPU composites. Viability of *E. coli* (**A**) and *S. aureus* (**B**) was evaluated at different bacterial culture densities after 24 h culture period. Data are presented as viability percentage to TPU set equal to 100%.

To evaluate the antibacterial properties of the TPU and TPU-based composites, the film was cut, sterilized, and rinsed. Bacterial suspensions of *E. coli* ATCC 25922 and *S. aureus* ATCC 25923 diluted at the indicated concentrations were dispensed on top of TPU materials and incubated at 37 °C for 24 h. To evaluate viability, MTT metabolic test was conducted and results are shown in Figure 13.

In general, the antibacterial effect of metals is mainly observed on Gram-negative bacteria [11,12,64] owing to their membrane structure. Unexpectedly, no decrease in viability in *E. coli* was detected at all culture densities used and for all TPU composites analyzed (Figure 13A). Conversely, *S. aureus* growth was efficiently inhibited at a percentage ranging from 20% to more than 50% (Figure 13B). Unexpectedly, no decrease in viability in *E. coli* was detected at all culture densities used and for all TPU composites analyzed (Figure 13A). The antibacterial effect of metals is mainly observed on Gram-negative bacteria [11,12,64] owing to their membrane structure. However, in this case, no effect was detectable. Instead, *S. aureus* growth was efficiently inhibited at a percentage ranging from 20% to 50% (Figure 13B). Gram-positive bacteria have a thick cross-linked peptidoglycan layer as the outermost part of the cell wall. Therefore, the eventual adhesion of Gram-positive bacteria to a surface is dominated by electrostatic and hydrophobic interactions. TPU, due to its polymeric nature, together with the employed fillers present the required characteristics that might endow the generated composites with superior selectivity for Gram-positive over Gram-negative bacteria. Furthermore, Gram-positive bacteria have been model organisms to study bacterial adhesion owing to the plethora of cell-wall anchored (CWA) proteins that they express [65].

These proteins mediate avid adhesion to surfaces and extracellular matrix components. Taken together these observations may explain the different antibacterial behavior displayed against Gram-positive and Gram-negative strains. In most cases, no significant difference was noticed at different bacterial culture densities. Only a modest correlation in the case of TPU-TiO_2_ was revealed, where higher inocula corresponded with lower inhibition levels.

**Figure 14:**
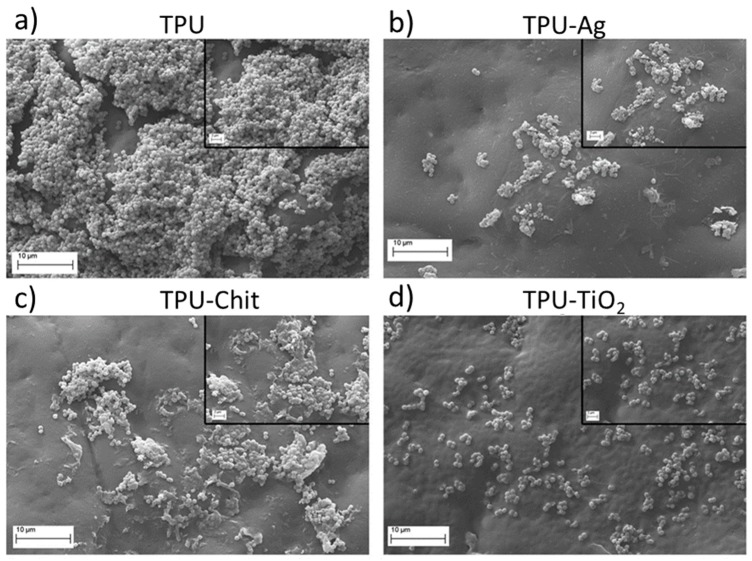
SEM micrographs of *S. aureus* ATCC25923 cultured on TPU (**a**), TPU-Ag (**b**), TPU-Chit (**c**), and TPU-TiO_2_ (**d**). Main micrograph: magnification 5000×, scale bar 10 μm, inset: magnification 10,000×, scale bar 2 μm.

To visualize if bacterial cell morphology or adhesion properties was hindered, observation were made of infected TPU and TPU-filled films with a SEM microscope. Figure 14 shows micrographs of the generated materials after infection with *S. aureus*. Although the levels of adhesion and growth on neat TPU were abundant and normal, a number of cells adhered in a more scattered fashion, compatible with a reduced growth and impaired adhesion on all the TPU composites. Furthermore, staphylococcal cells roundness is affected indicating cell-wall damage and disruption.

## 4. Conclusions

In this paper, TPU-based composites were successfully prepared and investigated. Antibacterial agents were directly incorporated into the main constituent material by simply melt compounding under the same processing conditions as the homopolymer. A molecular weight reduction was observed following the mixing treatment, which can be associated with the shortening of the polyurethane chains, associated with hydrolysis due to some water absorption during the process. The presence of the fillers affects the molecular weights, especially for TPU-TiO_2,_ due to the photo catalytic activity of titanium dioxide, in the form of anatase. TGA experiments confirmed that the presence of TiO_2_ influences the stability of the polymer chains and the initial degradation temperature was registered with a decrease of about 30 °C compared to the homopolymer. Considering that the first degradation stage is ascribed to the decomposition of the hard segments, the registered trend suggests a hard domains reduction, which is consistent with the results of the frequency sweep tests presented. Similarly, DSC data confirmed the crystallization of short-range disordered structures in presence of the fillers, which exhibited a melting peak at around 60 °C.

The complex TPU microphase separation, known for its strong dependence on TPU microstructure and thermal history, was even more affected by the fillers. By recording the storage modulus and the loss modulus vs. frequency, we observed a crossover shift to higher angular frequencies of TPU composites compared to the homopolymer typical of smaller hard domains for which shorter relaxation times are required to respond to the applied solicitation. Since the hydrogen bonding efficiency is perturbed by the filler presence and the hard domains size reduced, both the crystallization and melting of these domains are affected as a consequence of less well-defined crystals obtained. This is consistent with the X-ray diffraction experiments for which it appears that the presence of ordered structures in a predominantly amorphous matrix is influenced by the use of fillers. Deviations from the homopolymer rheological and mechanical behavior are registered for the composites and TiO_2_ affects the most this behavior.

However, all the fillers employed promote antibacterial activity. SEM images and their corresponding elemental analysis map showing the relative position of Ag and TiO_2_, highlight a homogeneous distribution of the fillers on the polymeric surfaces, which ensures such a capability of these materials. In particular, the best antibacterial effects were obtained with all the composites against *Staphylococcus aureus*. The TPU composites presented were able to kill this bacterium at four different bacterial culture concentrations.

Finally, we conclude that silver and chitosan can be easily used as fillers for medical devices since their impact on the thermal, mechanical, and morphological properties of TPU is limited. Their introduction as fillers did not require variations of the processing line neither for the processing parameters. TPU loss of crystalline ordered structures observed when TiO_2_ is used and the consequent embrittlement of TPU-TiO_2_ composite is an indication that such a filler is not suitable for catheters or other medical devices with similar applications.

## Figures and Tables

**Table 1:** Molecular and TGA data of the investigated materials.

Sample	*M*_w_ (Kg/mol)	*M*_n_ (Kg/mol)	*M*_w_ */M*_n_	*T*_2%_ (°C)	*T*_50%_ (°C)
TPU unprocessed	94.8	52.5	1.8		
TPU	80.7	47.3	1.7	309	386
TPU-Ag	76.7	45.0	1.7	305	385
TPU-Chit	77.6	45.4	1.7	305	379
TPU-TiO_2_	51.0	33.8	1.5	279	383

*M*_w_ = weight-average molecular weight; *M*_n_ = number-average molecular weight from SEC; *T*_2%_ = temperature at which the initial 2% mass loss occurs; *T*_50%_ = temperature corresponding to 50% mass loss from TGA.

**Table 2:** Uniaxial tensile data for TPU and TPU-based composites.

Sample	*E* (MPa)	*σ*_max_ (MPa)	*ε*_break_ (%)
TPU	26.2 ± 1.4	36.4 ± 1.6	1075 ± 44
TPU-Ag	30.5 ± 1.6	26.4 ± 3.1	975 ± 87
TPU-chitosan	33.9 ± 1.5	25.9 ± 1.3	845 ± 21
TPU-TiO_2_	19.8 ± 4.7	4.4 ± 1.1	123 ± 45

*E =* elastic modulus, *σ*_max_ = maximum stress, and *ε*_break_ = elongation at break.

**Figure A1:**
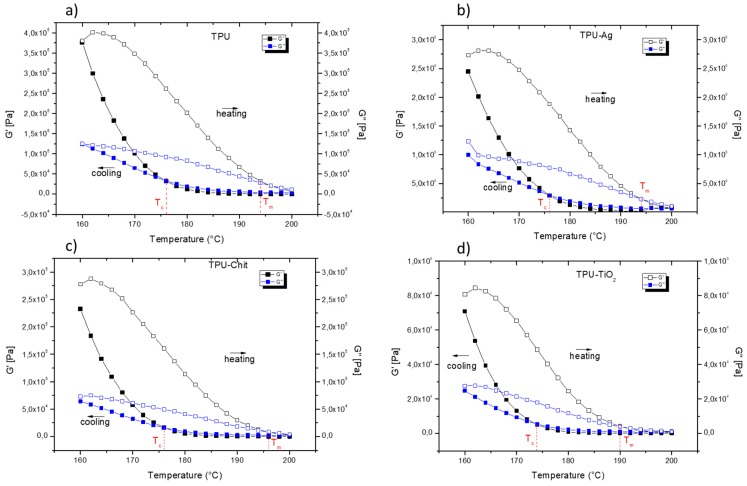
Storage modulus and loss modulus at 10 rad/s as a function of the temperature range T = 160–200 °C, on cooling (filled symbols) and on heating (open symbols) for TPU (**a**), and TPU-Ag (**b**), TPU-Chit (**c**), and TPU-TiO_2_ (**d**).

## Supplementary Materials

The following are available online at https://www.mdpi.com/2073-4360/12/2/362/s1. Part S1: NMR signal assignment and structure correlation; Part S2: FTIR peak assignment; Figure S1: FTIR spectra of the TPU and TPU-based composites; Figure S2: DSC traces (I heating) of TPU and TPU-based composites; Figure S3: Surface morphology images of TPU and TPU-based composites; Figure S4: Time sweep experiments at 190 °C and different angular frequencies (ω = 0.1 rad/s, ω = 1 rad/s and ω = 10 rad/s) for TPU (a) and a comparison between TPU and TPU-TiO_2_ behavior measured at ω = 0.1 rad/s (b); Figure S5: Storage modulus at 10 rad/s as a function of the temperature, on cooling (filled symbols) and on heating (open symbols) for TPU and TPU-based composites; Table S1: Composition detected by EDS analysis of TPU-Ag and TPU-TiO_2_ film.

## Appendix A

The temperature sweep experiments were performed at 10 rad/s for TPU and TPU composites during cooling from 200 to 160 °C (filled symbols) and heating vice versa (open symbols) and reported in Figure A1.

As already mentioned in this paper, during cooling, the storage modulus (G’) of each of these samples increases rapidly due to the crystallization of the more ordered hard domains typical of the TPU matrix. On the contrary, G’ decreases on heating due to these domains melting at a temperature higher than they form on cooling. By plotting G’ and G’’ collected during the temperature sweep experiments, it is possible to determine the crystallization and melting temperatures registered for these heating/cooling cycles, which are given by the crossover points between G’ and G”. On cooling, when G’ crosses G’’ before becoming dominant (G’ > G”) the crystallization of these domains starts. The mentioned crossover, which occurs at 176 °C i.e., for TPU, represents the crystallization temperature of these domains [65]. Analogously, the crossover registered on heating, when G” crosses G’, represents the melting temperature of the hard domains. By comparing the hysteresis behavior registered for all the samples studied and the range of values respectively associated, a reduction of the areas enclosed by G’ curves (registered on heating and on cooling) is observed. We believe these results strengthen what already described in the paper regarding G’ deviation. Both crystallization and melting are affected by the fillers presence compared to TPU as effect of the reduced and less defined crystals obtained (Table A1). TPU-TiO_2_ affects the most this behavior and a variation of *T*_c_ and *T*_m_ collected from the temperature sweep experiments is registered. Although these values could be influenced by a heating/cooling rate (~0.7 °C/min) which depends on the instrumental acquisition time of each single point, it is evident that the TPU-TiO_2_ is the sample for which dimensions and shape of these hard domains are affected the most.

**Table A1:** *T*_c_ and *T*_m_ from temperature sweep experiments.

Sample	*T_c_* (°C)	*T*_m_ (°C)
TPU	176	194
TPU-Ag	176	194
TPU-Chit	176	194
TPU-TiO_2_	174	190

*T*_c_: crystallization temperature; *T*_m_: melting temperature.
