# Supplementary material for: Polyurethane-Based Composites: Effects of Antibacterial Fillers on the Physical-Mechanical Behavior of Thermoplastic Polyurethanes
Source: Polymers (Basel). 2020 Feb 6;12(2):362. doi: 10.3390/polym12020362 (PMC7077423; doi:10.3390/polym12020362)
Supplement: Supplementary file 1 [file polymers-12-00362-s001.pdf]

Supplementary material

Polyurethane-based Composites: Effects of Antibacterial Fillers on the Physical-Mechanical Behaviour of Thermoplastic Polyurethanes

Maurizio Villani [1,*], Roberto Consonni [1], Maurizio Canetti [1], Federico Bertoglio [2,3,4] , Stefano Iervese [2], Giovanna Bruni [5], Livia Visai [2,4] , Salvatore Iannace [6] and Fabio Bertini [1,*]

[1] Istituto di Scienze e Tecnologie Chimiche "Giulio Natta" – CNR, Via A. Corti 12, 20133 Milano, Italy; roberto.consonni@scitec.cnr.it (R.C.); maurizio.canetti@scitec.cnr.it (M.C.)

[2] Department of Molecular Medicine (DMM), Center for Health Technologies (CHT), UdR INSTM, University of Pavia, Viale Taramelli 3/B, 27100 Pavia, Italy; federico.bertoglio01@ateneopv.it (Fe.B.); stefano.iervese01@ateneopv.it (St.I.); livia.visai@unipv.it (L.V.)

[3] School for Advanced Studies IUSS, Palazzo del Broletto Piazza della Vittoria, 15, 27100 Pavia, Italy; federico.bertoglio@iusspavia.it (Fe.B.)

[4] Department of Occupational Medicine, Toxicology and Environmental Risks, Istituti Clinici Scientifici Maugeri S.p.A Società Benefit, IRCCS, Via S. Boezio, 28, 27100 Pavia, Italy; livia.visai@fsm.it (L.V.)

[5] Department of Chemistry, Physical Chemistry Section, University of Pavia, viale Taramelli 16, 27100 Pavia, Italy; giovanna.bruni@unipv.it (G.B.)

[6] Istituto per i Polimeri, Compositi e Biomateriali – CNR, Piazzale Enrico Fermi 1, 80055 Portici (NA), Italy; iannace@unina.it (Sa.I.)

* Correspondence: maurizio.villani@scitec.cnr.it (M.V.); fabio.bertini@scitec.cnr.it (Fa.B.)

Part S1: NMR signal assignment and structure correlation. NMR spectra recorded on neat TPU are reported in Figure 2. 1H-NMR spectrum showed the typical signal pattern of the aromatic MDI (4,4′ di-phenylmethane diisocianate) typically linked with a diol moiety. 1H-NMR signals of the aromatic protons (7.30 and 7.12 ppm) confirmed the 4,4′-di-substitution of the diphenylmethane; furthermore signal at 3.89 ppm, showed the integral ratio compatible with a single benzylic moiety, also confirmed by the ratio with the integral of signal at 6.77 ppm due to two amide protons. Integral values of methylene groups centred at 4.19 ppm, due to the -CH2OCONH- moiety, signals at 4.09 ppm due to -OCOCH2- and integrals of signals centred at 1.74 ppm due to -CH2- moiety confirmed the presence of a diol moiety (butanoic), with additional signals occurring at 2.34 ppm, due to -CH2COO- moiety. Bidimensional heteronuclear 1H-13C experiment (HMBC) highlighted structural details. In particular, a typical carboxylic carbon signal (at 172.7 ppm) revealed a long range correlation with proton signal at 2.34 ppm due to –CH2COO- moiety, with signal at 4.09 ppm due to -OCOCH2- moiety and with signals at 1.74 ppm due to the -CH2- moiety; those correlations, in combination with 1H integral values of corresponding protons, indicated the presence of an adipic moiety linking to butanediol moieties. The other carbonylic signal due to amide moiety was occurring at 153.4 ppm showing a long range correlation only with the -CH2OCONH- moiety at 4.19 ppm. Aromatic carbons occurred at 119.17 and 129.22 ppm, quaternary aromatic carbons at 135.88 and 136.24 ppm; and benzylic methylene at 40.41 ppm. All other methylene carbons occurred at about 24 ppm.

Part S2: FTIR peak assignment. Fourier Transform Infrared (FTIR) spectra were acquired in attenuated total reflectance (ATR) mode, in the spectral range of 4000–450 cm^{-1}, using a Perkin Elmer (Waltham, MA, USA) Spectrum Two spectrophotometer equipped with a diamond ATR crystal. Figure S1 shows FTIR spectra of the investigated materials recorded at room temperature and normalized according to the area under the symmetric and asymmetric methylene bands in the interval 3000-2800 cm^{-1}. No significant differences among the prepared composites with different antibacterial filler and the neat TPU have been observed. All the signals are characteristics of polyurethane-based materials. In particular, the spectra show a signal related to NH stretching in the range of 3300-3000 cm^{-1}, the symmetric and asymmetric methylene bands at 3000-2800 cm^{-1} , the CO stretching of urethane groups and hydrogen bonded urethane groups (Amide I, 1730-1700 cm^{-1}), C=C skeletal stretch of benzene ring (1597 cm^{-1}), in-plane NH deformation with CO and CN stretches (Amide II, 1529 cm^{-1}), CN stretches and C-O-C stretches.

Since a FTIR spectral imaging was not available, several measurements were performed on different areas of the samples in order to look for possible variations of hydrogen bonded/free carbonyl balance of each composite prepared as an indication of possible hydrogen bonding perturbation due to the fillers presence. Unfortunately, no appreciable differences were observed.

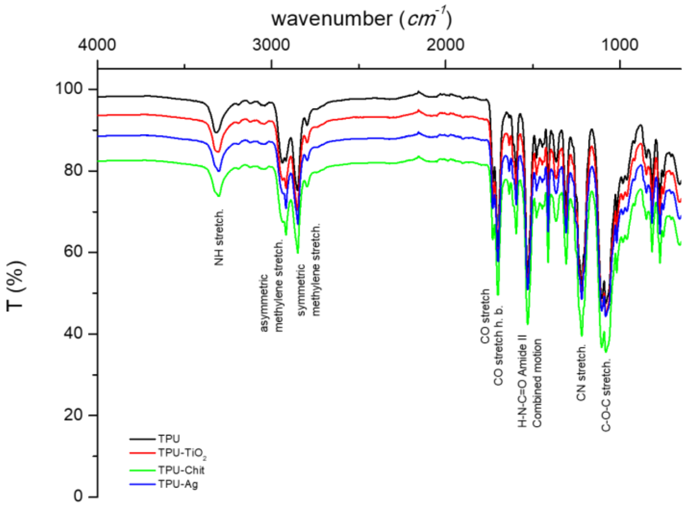

Figure S1. FTIR spectra of the TPU and TPU-based composites.

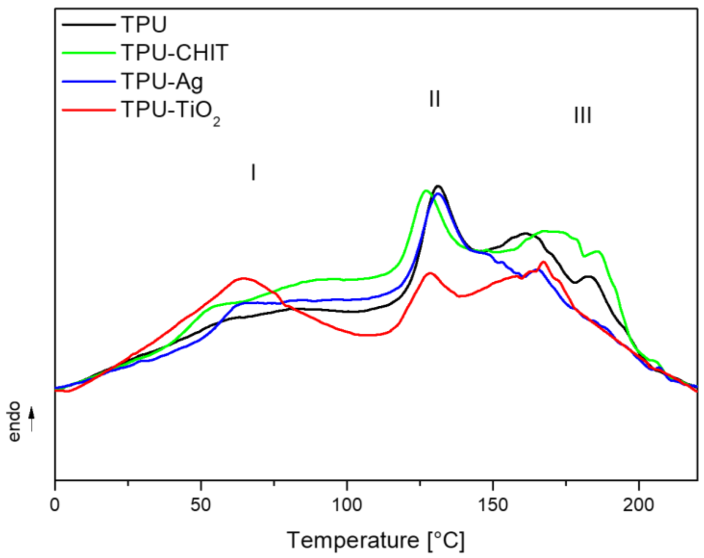

Figure S2. DSC first heating traces of TPU and TPU-based composites.

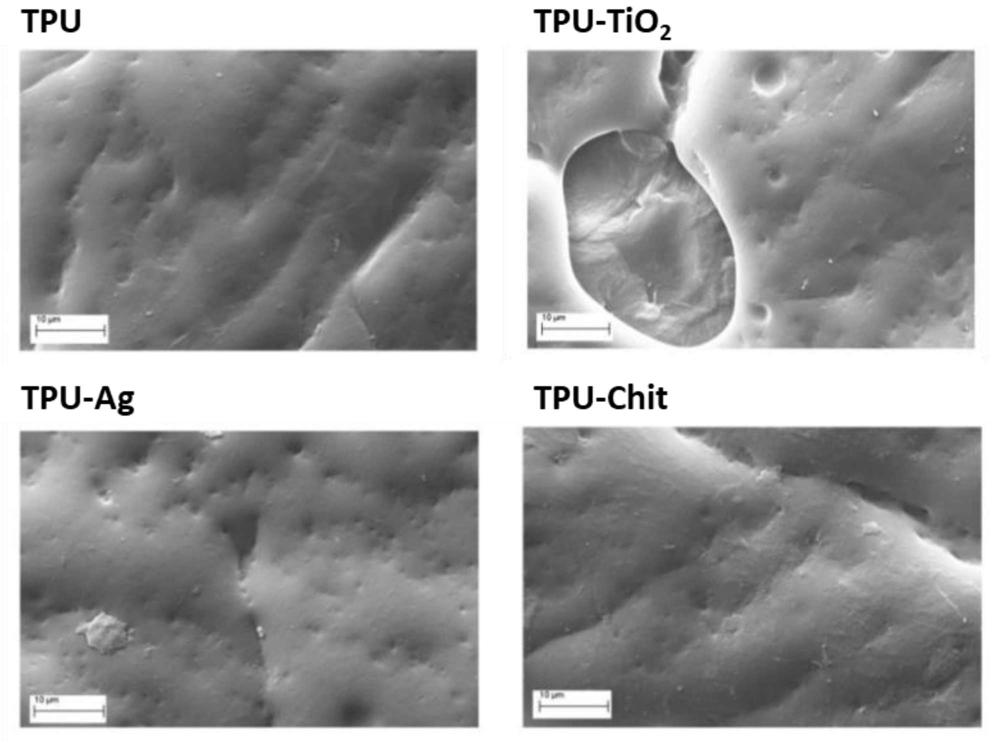

Figure S3. Surface morphology images of TPU (a) and TPU-based composites (b-d). Scale bars represent 10μm.

Table S1. Summary of composition detected by EDS analysis of TPU-Ag and $TPU-TiO_2$ films.

	TPU-Ag		$TPU-TiO_2$	
**Element**	**Weight %**	**Weight % Sigma**	**Weight %**	**Weight % Sigma**
C	73.25	0.24	73.01	0.23
O	26.50	0.24	26.36	0.23
Ag	0.25	0.03		
Ti			0.63	0.02
Total	100.00		100.00	

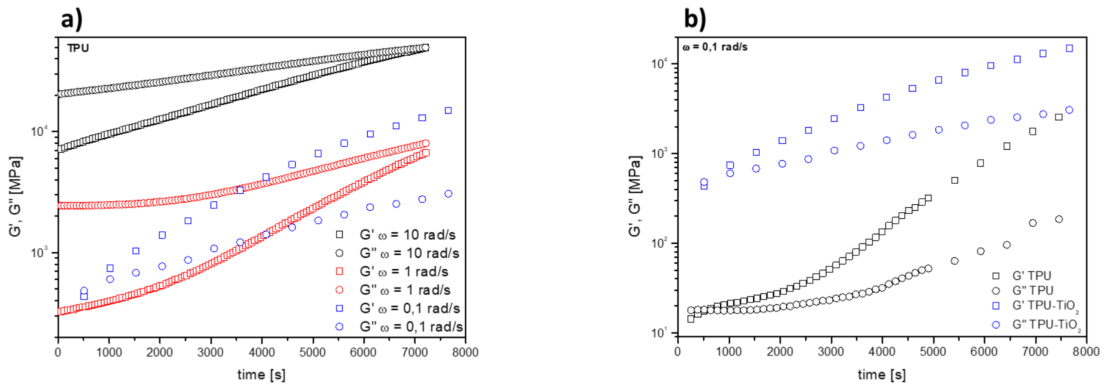

Figure S4. Time sweep experiments at 190 °C and different angular frequencies (ω = 0.1 rad/s, ω = 1 rad/s and ω = 10 rad/s) for TPU (a) and a comparison between TPU and $TPU-TiO_2$ behaviour measured at ω = 0.1 rad/s (b).

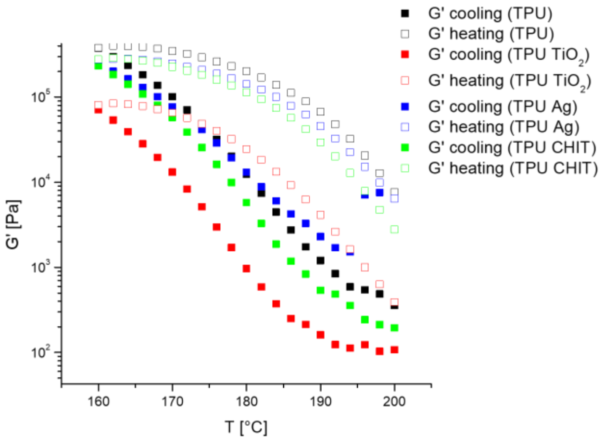

Figure S5. Storage modulus at 10 rad/s as a function of the temperature (T = 160-200 °C), on cooling (filled symbols) and on heating (open symbols) for TPU and TPU-based composites.
